# The Neuropsychiatric Disease-Associated Gene *cacna1c* Mediates Survival of Young Hippocampal Neurons^1^,^2^,^3^

**DOI:** 10.1523/ENEURO.0006-16.2016

**Published:** 2016-03-31

**Authors:** Anni S. Lee, Héctor De Jesús-Cortés, Zeeba D. Kabir, Whitney Knobbe, Madeline Orr, Caitlin Burgdorf, Paula Huntington, Latisha McDaniel, Jeremiah K. Britt, Franz Hoffmann, Daniel J. Brat, Anjali M. Rajadhyaksha, Andrew A. Pieper

**Affiliations:** 1 Feil Family Brain and Mind Research Institute, Weill Cornell Medicine, Cornell University, New York, New York 10065; 2 Division of Pediatric Neurology, Department of Pediatrics, Weill Cornell Medicine, Cornell University, New York, New York 10065; 3 Neuroscience Graduate Program, UT Southwestern Medical Center, Dallas, Texas 75390; 4 Department of Psychiatry, University of Iowa, Carver College of Medicine, Iowa City, Iowa 52242; 5 Department of Psychiatry, UT Southwestern Medical Center, Dallas, Texas 75390; 6 Institute of Pharmacology, Technical University Munich, Munich, Germany; 7 Research Group 923, Technical University Munich, Munich, Germany; 8 Pathology and Laboratory Medicine, Emory University School of Medicine, Atlanta, Georgia 30322; 9 Weill Cornell Autism Research Program, Weill Cornell Medical College, New York, New York 10065; 10 Department of Neurology, University of Iowa, Carver College of Medicine, Iowa City, Iowa 52242; 11 Department of Free Radical and Radiation Biology Program, Department of Radiation Oncology Holden Comprehensive Cancer Center, University of Iowa, Carver College of Medicine, Iowa City, Iowa 52242; 12 Department of Veteran Affairs, University of Iowa Carver College of Medicine, Iowa City, Iowa 52242

**Keywords:** anxiety, Cav, neurogenesis, neuroprotection, P7C3, P7C3A20

## Abstract

Genetic variations in *CACNA1C*, which encodes the Ca_v_1.2 subunit of L-type calcium channels (LTCCs), are associated with multiple forms of neuropsychiatric disease that manifest high anxiety in patients. In parallel, mice harboring forebrain-specific conditional knockout of *cacna1c* (forebrain-Ca_v_1.2 cKO) display unusually high anxiety-like behavior. LTCCs in general, including the Ca_v_1.3 subunit, have been shown to mediate differentiation of neural precursor cells (NPCs). However, it has not previously been determined whether Ca_v_1.2 affects postnatal hippocampal neurogenesis *in vivo*. Here, we show that forebrain-Ca_v_1.2 cKO mice exhibit enhanced cell death of young hippocampal neurons, with no change in NPC proliferation, hippocampal size, dentate gyrus thickness, or corticosterone levels compared with wild-type littermates. These mice also exhibit deficits in brain levels of brain-derived neurotrophic factor (BDNF), and Cre recombinase-mediated knockdown of adult hippocampal Ca_v_1.2 recapitulates the deficit in young hippocampal neurons survival. Treatment of forebrain-Ca_v_1.2 cKO mice with the neuroprotective agent P7C3-A20 restored the net magnitude of postnatal hippocampal neurogenesis to wild-type levels without ameliorating their deficit in BDNF expression. The role of Ca_v_1.2 in young hippocampal neurons survival may provide new approaches for understanding and treating neuropsychiatric disease associated with aberrations in *CACNA1C*.

Visual Abstract

## Abstract (precis)

Genetic variations in *CACNA1C*, which encodes the Ca_v_1.2 subunit of L-type calcium channels (LTCCs), are associated with multiple forms of neuropsychiatric disease that manifest high anxiety in patients.

## Visual Overview

**Figure:**
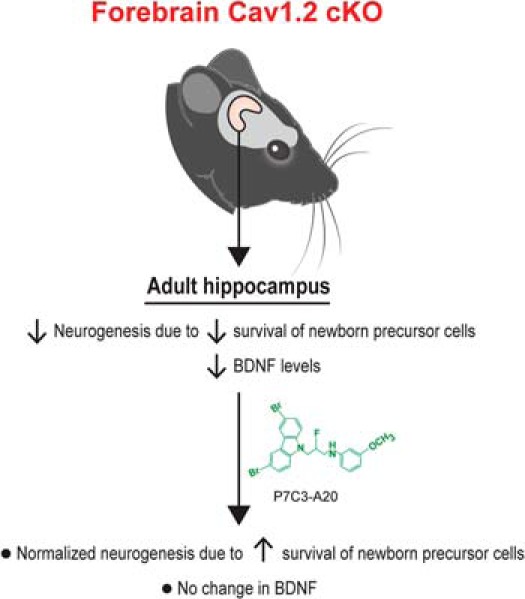

## Significance Statement

Aberrant postnatal hippocampal neurogenesis and *CACNA1C* mutations are associated with neuropsychiatric diseases manifesting high anxiety, and mice deficient in Ca_v_1.2 neuronal expression display high anxiety-like behavior. Here, we report that these mice also display deficient postnatal hippocampal neurogenesis by virtue of elevated death of young hippocampal neurons, along with decreased expression of the endogenous proneurogenic agent brain-derived neurotrophic factor (BDNF). We further show that treatment of these mice with the neuroprotective agent P7C3-A20 circumvents the BDNF deficiency to safely and effectively normalize hippocampal neurogenesis without altering BDNF levels. Pharmacologic agents derived from the P7C3 family of neuroprotective compounds could thus provide a new therapeutic approach for treating patients suffering from neuropsychiatric disease associated with aberrations in *CACNA1C*.

## Introduction

*CACNA1C* is one of the most widely reproduced risk genes for neuropsychiatric disorders (Heyes et al., 2015), including bipolar disorder (Ferreira et al., 2008; Sklar et al., 2008; Green et al., 2010, 2013; Lee et al., 2011; Psychiatric GWAS Consortium Bipolar Disorder Working Group, 2011; Nurnberger et al., 2014; Ament et al., 2015), schizophrenia (Nyegaard et al., 2010; Hamshere et al., 2013; Ripke et al., 2013; Schizophrenia Working Group of the Psychiatric Genetics Consortium, 2014) , and major depressive disorder (Casamassima et al., 2010; Green et al., 2010). *CACNA1C* was also recently identified in the largest human genome-wide association study to date as one of only two genes presenting a common risk factor across five major forms of neuropsychiatric illness: major depression, schizophrenia, bipolar disorder, autism, and attention deficit hyperactivity disorder (ADHD; Cross-Disorder Group of the Psychiatric Genomics Consortium, 2013). It is not known, however, how *CACNA1C* exerts such pleiotropic effects on psychopathology.

*CACNA1C* encodes the voltage-gated L-type calcium channel (LTCC) Ca_v_1.2, which allows cellular influx of calcium following transient changes in membrane potential. This ultimately activates downstream pathways of genetic transcription, such as for brain-derived neurotrophic factor (BDNF; Ghosh et al., 1994; Tao et al., 1998). Ca_v_1.2 also plays an important role in synaptic plasticity related to neuropsychiatric illness and drug addiction (Giordano et al., 2010; Schierberl et al., 2011), reward-driven behavior (Wessa et al., 2010; Lancaster et al., 2014), fear conditioning (White et al., 2008; Langwieser et al., 2010), and cognition (Moosmang et al., 2005; White et al., 2008). Furthermore, Ca_v_1.2, and not the other brain-specific LTCC subunit Ca_v_1.3, mediates anxiety-like behavior in mice (Dao et al., 2010; Lee et al., 2012). Specifically, mice harboring forebrain-specific conditional knockout of *cacna1c* (forebrain-Ca_v_1.2 cKO) show elevated anxiety-like behavior in the light/dark conflict test, the open-field test, and the elevated plus maze (Lee et al., 2012). Notably, anxiety is a prominent component of all forms of neuropsychiatric illness in which *CACNA1C* has been implicated.

Deisseroth et al. (2004) have previously shown a bidirectional regulatory role of LTCCs in adult-derived neural precursor cell proliferation *in vitro*, and Ca_v_1.3 has recently been demonstrated to modulate both proliferation of postnatal neural precursor cells (NPCs) and survival of young hippocampal neurons in the hippocampus, such that elimination of Ca_v_1.3 results in reduced size of the dentate gyrus (Marschallinger et al., 2015). This effect was related to expression of Ca_v_1.3 in both immature NPCs (Nestin-positive) and mature (NeuN-positive) young hippocampal neurons, whereas Ca_v_1.2 expression is restricted to only mature young hippocampal neurons (Marschallinger et al., 2015) in adult mice. However, it has not previously been determined whether Ca_v_1.2 exerts a unique or complementary role in LTCC-mediated hippocampal neurogenesis, the net magnitude of which is a balance of proliferation of NPCs and survival of young hippocampal neurons into which NPCs differentiate. We sought to address this question because of the role of postnatal hippocampal neurogenesis in the broad spectrum of neuropsychiatric diseases in which aberrations in both *CACNA1C* (as described above) and postnatal hippocampal neurogenesis have been implicated, including major depression (Serafini et al., 2014; Walker et al., 2015), schizophrenia (Pieper et al., 2005; Pickard et al., 2006; Reif et al., 2007; Le Strat et al., 2009; Pickard 2011; Wu et al., 2013; Schreiber and Newman-Tancredi, 2014), bipolar disorder (Knight et al., 2012; Nurnberger et al., 2014; Takamura et al., 2014), autism (Amiri et al., 2012; Singh et al., 2013; Stanco et al., 2014), and ADHD (Dabe et al., 2013; Jolly et al., 2013; Ohira et al., 2013; Kobayashi et al., 2014). Specifically, we applied forebrain-Ca_v_1.2 conditional deletion (cKO), as well as viral vector-mediated *cacna1c* gene elimination in adult mice, to quantify hippocampal neurogenesis and other neurophysiologic parameters following spatial and temporal manipulation of Ca_v_1.2 expression.

## Materials and Methods

### Animals

All animal procedures were performed in accordance with the University of Iowa, Weill Cornell Medical College, and UT Southwestern animal care committee’s regulations. Animals were housed in temperature-controlled conditions, provided food and water *ad libitum*, and maintained on a 12 h light/dark cycle (7:00 A.M. to 7:00 P.M.). Male C57BL/6J mice were purchased from The Jackson Laboratory. Forebrain- Cav1.2 cKO mice were generated by crossing homozygous *cacna1c* (Ca_v_1.2) floxed mice (*cacna1c* ^fl/fl^; Moosmang et al., 2005) with mice expressing Cre recombinase under the control of the alpha-CaMKII promoter (CaMKII-Cre). The CaMKII-Cre T29-1 line from Jackson Laboratories was used. In this line, Cre expression is activated at postnatal day (P)18, thereby circumventing early developmental compensatory adaptations. *HET*s and forebrain- Cav1.2 cKO were indistinguishable from wild-type (WT) in weight, development, and general health.

### BrdU staining

After BrdU (Sigma-Aldrich) administration, mice were euthanized at the described time points by transcardial perfusion with 4% paraformaldehyde at pH 7.4 and brains were processed for immunohistochemical detection of incorporated BrdU in the hippocampus. Dissected brains were immersed in 4% paraformaldehyde overnight at 4°C, and then cryoprotected in sucrose before being sectioned into 40-μm-thick free-floating sections. Unmasking of BrdU antigen was achieved through incubating tissue sections for 2 h in 50% formamide/2× saline-sodium citrate (SSC) at 65°C, followed by a 5 min wash in 2× SSC and subsequent incubation for 30 min in 2 m HCl at 37°C. Sections were processed for immunohistochemical staining with mouse monoclonal anti-BrdU (1:100, Roche). The number of BrdU+ cells in the entire dentate gyrus subgranular zone (SGZ) was quantified by counting BrdU+ cells within the SGZ and dentate gyrus in every fifth section throughout the entire hippocampus, and then normalizing for dentate gyrus volume using Nikon Metamorph and NIH ImageJ software with appropriate conversion factors.

### Surgery

Anesthesia was induced by intraperitoneal injection of ketamine (100 mg/kg)/xylazine mixture (10 mg/kg). A midline incision was made, local anesthesia (Marcaine) applied, the head leveled, and holes formed through the skull using a 25 gauge needle. Region-specific deletion of *cacna1c* was generated by manual bilateral infusion of AAV2/2-Cre-GFP (Vector BioLabs; 0.75 μl/side) into the hippocampus of cacna1c^floxed/floxed^ mice through a 2.5 μl Hamilton syringe at a rate of 0.1 μl/min. AAV2/2-GFP (Vector BioLabs) was used as a control. The coordinates for the hippocampus were as follows: anterior–posterior −2 mm; media–lateral ±1.6 mm; dorsal–ventral −1.8 mm, at a 10°angle. The needle was held in place for an additional 5 min after infusion to ensure complete delivery of virus. After a minimum of 3 weeks to allow for maximal Cre recombinase expression, mice were administered 50 mg/kg BrdU for 5 d and transcardially perfused with 4% paraformaldehyde (PFA) 24 h after the last injection of BrdU.

### Fluorescent immunohistochemistry

Ca_v_1.2 fluorescent immunohistochemistry was performed to confirm elimination of Ca_v_1.2. Fluorescent immunohistochemistry was also used to confirm injection placement. Mice were transcardially perfused with 4% PFA, and brains were dissected and postfixed overnight in 4% PFA followed by cryoprotection in 30% sucrose at 4°C for at least 72 h. Forty-micrometer-thick sections spanning the hippocampus were obtained using a sliding microtome and incubated in anti-chicken GFP (1:10,000, Aves Labs) and anti-rabbit glial fibrillary acidic protein (1:1000, Invitrogen) primary antibody overnight at 4°C. Sections were rinsed in 0.1 m phosphate-buffer (PB) and incubated with donkey AlexaFluor 488 (1:300) and AlexaFluor 568 (1:300) antibody for 1 h at room temperature. Doublecortin fluorescent immunohistochemistry was performed to analyze cells in the dentate gyrus that had recently committed to neuronal fate. Sections were incubated in anti-guinea pig doublecortin (1:5000, Millipore) primary antibody overnight at 4°C. Sections were rinsed in 0.1 m PB and incubated with donkey AlexaFluor 594 (1:400) antibody for 1 h at room temperature. Sections were imaged using an epifluorescent microscope (Leica DM550B with Leica Application Suite Advanced Fluorescence 3.0.0 build 8134 software, Leica Microsystems).

### q-PCR

To measure doublecortin (*DCX*) mRNA levels in forebrain Ca_v_1.2 cKO mice and AAV2-2/2-Cre-GFP injected *cacna1c floxed* (*cacna1c* ^fl/fl^) mice, mice were euthanized by rapid decapitation and whole brains were rapidly dissected. Brain tissue was sectioned on a 1 mm brain block. Dentate gyrus-containing tissue punches were obtained from forebrain Cav1.2 cKO and wild-type mice. For AAV2/2-Cre-GFP and AAV2/2-GFP injected mice, GFP goggles (BLS) were used to visualize GFP signal in brain sections containing the dentate gyrus and to selectively dissect GFP-positive tissue. Tissue punches were processed for total RNA isolation using the mirVana RNA isolation kit (Life Technologies) and cDNA was synthesized from purified RNA using the High Capacity RNA-to-cDNA kit (Applied Biosystems). Cav1.2 mRNA levels were measured using *cacna1c*-specific primers (Qiagen QuantiTect Primer assay QT00150752), and *DCX* levels were measured using *DCX*-specific primers (Qiagen QuantiTect Primer assay QT02521155) on an ABI PRISM 7000 Sequence Detection System with SYBR Green PCR Master Mix (Applied Biosystems). Cycle threshold (*C*t) values for target genes were normalized to the housekeeping gene *gapdh* (QuantiTect Primer assay QT01658692, Qiagen). Each experiment was performed in triplicate and values were averaged.

### BDNF ELISA

Mature BDNF protein level was measured using the BDNF Emax ImmunoAssay (ELISA) system (Promega), with recombinant mature BDNF as a standard. Standard and samples were performed in duplicate, with each group containing 10–14 samples. Protein was extracted and quantified following the manufacturer’s protocol. Tissue samples were homogenized in lysis buffer (150 mm NaCl, 1% Triton X-100, 25 mm HEPES, 2 mm NaF) containing phosphatase and protease inhibitors, and then incubated by rotation at 4°C for 1 h. Homogenized tissue was centrifuged at maximum speed and the supernatant containing total protein was collected and quantified using the BCA protein assay kit (Thermo Fisher Scientific). Each sample was diluted 1:1 with block and sample buffer (BSB), and placed in designated wells of a 96-well plate previously coated with BDNF antibody in carbonate buffer (25 mm Na_2_CO_3_ and 25 mm Na_2_HCO_3_, pH 9.7, incubated at 4°C), followed by blocking with BSB. A second coating of primary anti-human BDNF antibody was added, followed by horseradish peroxidase-conjugated secondary antibody. The colorimetric reaction was initiated by tetramethylbenzidine. After 10 min, the reaction was stopped by addition of 1N HCl, and absorbance was read at 450 nm on a plate reader (iMark Absorbance Microplate Reader, Bio-Rad Laboratories).

### Corticosterone levels

To measure baseline and stress-induced corticosterone levels, plasma samples were isolated from 7- to 15-week-old forebrain-Ca_v_1.2 cKO and wild-type mice at 1:00–2:00 P.M. Plasma was isolated from trunk blood. Blood was allowed to sit at room temperature for 60 min and spun at 1200 × *g* for 15 min. Supernatant was isolated and stored at −20°C. For all restraint stress experiments, mice were restrained for 30 min in decapicones. Plasma corticosterone levels were measured using the high-sensitivity corticosterone enzyme immunoassay (EIA) kit (AC-15F1, Immunodiagnostic Systems). Samples were analyzed in duplicate. Concentrations were determined per the manufacturer’s instructions.

### Morphometric analysis of hippocampal size

Four percent paraformaldehyde-fixed mouse brains were sectioned in the coronal plane, paraffin-embedded, sectioned at 8-μm-thickness, and stained with hematoxylin & eosin. Histological sections were obtained at 50 mm intervals. Measurements of the hippocampus, dentate granular cell layer, and forebrain were taken at the coronal level in which CA1 approaches the midline and the upper blade of the dentate gyrus runs parallel to the surface of the brain. An ocular lens fitted with an etched grid was used to measure the dentate, CA1, and CA3 height and neuronal size (60×), as well as hippocampal dimensions (2×).

### P7C3-A20 treatments

All mice were single-housed for the duration of treatment. Forebrain-Ca_v_1.2 cKO and wild-type littermate mice received 10 mg/kg P7C3-A20 or vehicle (5% DMSO, 20% cremaphor in 5% dextrose), intraperitoneally, twice a day for 30 d, starting at P21. This dose of P7C3-A20 was chosen based on efficacy in multiple animal models of neuroprotection (De Jesus-Cortés et al., 2012; Tesla et al., 2012; Yin et al., 2014). Mice were transcardially perfused with 4% PFA 24 h after the last BrdU injection. In separate experiments, brains were flash frozen and processed for BDNF ELISA.

### Statistics

For all experiments, data were first analyzed for normality using a Shapiro–Wilk test. If the data were normally distributed, a parametric independent-samples *t* test or two-way ANOVA test was then applied. For data that were not normally distributed, a nonparametric independent-samples Mann–Whitney *U* test (as specified in figure legends), was applied. A value of *p* ≤ 0.05 was considered to be statistically significant and all analyses were performed using SPSS v19 (SPSS). Graphs were constructed in GraphPad Prism v6.0 for Macintosh.

## Results

### Ca_v_1.2 channels support postnatal hippocampal neurogenesis

**Figure 1.**
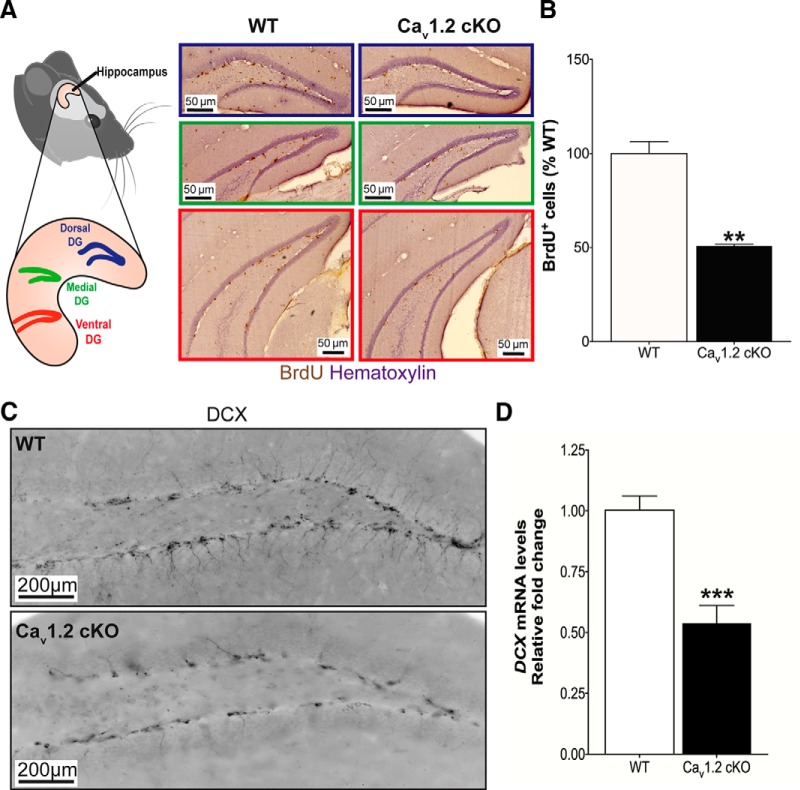
Ca_v_1.2 supports adult hippocampal neurogenesis. ***A***, Left, Graphical representation of the dorsal, medial, and ventral dentate gyrus (DG) in which BrdU^+^ staining was quantified. Right, Representative images of BrdU- and hematoxylin-stained DG from forebrain-Ca_v_1.2 cKO and WT littermate mice. ***B***, Forebrain-Ca_v_1.2 cKO mice show significantly lower BrdU^+^ cells in the DG compared with WT animals (***B***; WT, *n*= 4; KO, *n*=4; **p = 0.004, independent samples *t* test). ***C***, ***D***, Forebrain-Ca_v_1.2 cKO mice also show lower DCX protein (***C***; WT, *n*=3; KO, *n*=3) and mRNA levels (***D***; WT, *n*=6; KO, *n*=6; ***p < 0.001, independent samples t test) compared with WT animals. All graphs are represented as mean ± SEM.

To examine the net magnitude of adult hippocampal neurogenesis, which results from the balance of proliferation of NPCs and survival of young adult hippocampal neurons into which NPCs differentiate, in forebrain-Ca_v_1.2 cKO mice, all mice received intraperitoneal injections of the thymidine analog bromodeoxyuridine (BrdU, 50 mg/kg/d) once daily for 5 d. Mice were then euthanized for immunohistochemical analysis of the brain 24 h after the final BrdU injection. Compared to wild-type littermates, forebrain-Ca_v_1.2 cKO mice showed ∼50% fewer BrdU+ cells throughout the hippocampus (Fig. 1*A*,*B*; *F*_(1,7)_ = 57.714, *p* = 0.004). These mutant mice also exhibited significantly lower expression of doublecortin (Fig. 1*C*,*D*; *F*_(1,11)_ = 24.928, *p* < 0.001), a microtubule-associated protein that serves as a marker of neurogenesis by virtue of transient expression in newly formed neurons between their birth and final maturation (Brown et al., 2003).

**Figure 2.**
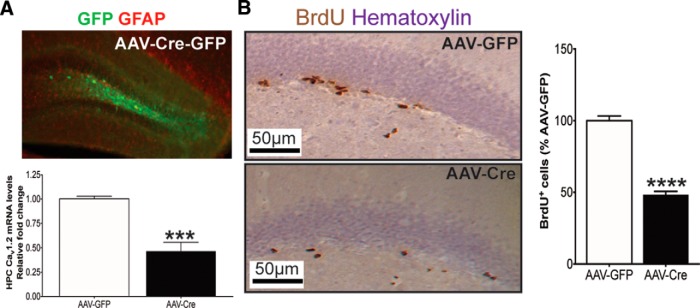
Selective elimination of Ca_v_1.2 in adult dentate gyrus results in lower neurogenesis. ***A***, Top, Representative image of GFP-labeled cells within the dentate gyrus of Ca_v_1.2^fl/fl^ mice injected with AAV2/2-Cre-GFP. Bottom, AAV2/2-Cre-GFP significantly decreased Ca_v_1.2 mRNA compared with control AAV2/2-GFP injected mice (***A***; AAV2/2-GFP, *n*=5; AAV2/2-Cre, *n*= 5; ***p < 0.001, independent samples *t* test)**. *B***, Adult focal hippocampal knockout of Ca_v_1.2 results in significantly lower BrdU^+^ cells in the dentate gyrus (AAV2/2-GFP, *n*=6; AAV2/2-Cre, *n*= 9; ****p < 0.0001, independent samples *t* test). All graphs are represented as mean ± SEM.

To directly evaluate the effect of spatially- and temporally-specific elimination of Ca_v_1.2 in the adult hippocampus, and thus differentiate between an adult versus developmental effect of Ca_v_1.2 on postnatal hippocampal neurogenesis, we next stereotaxically delivered AAV2/2-Cre-GFP into the dentate gyrus of adult *cacna1c* ^fl/fl^ mice. This resulted in significantly lower levels of Ca_v_1.2 mRNA compared to control AAV2/2-GFP injected mice (Fig. 2*A*; *F*_(1,9)_ = 31.536, *p* < 0.001). As with forebrain-Ca_v_1.2 cKO mice, focal knockout of Ca_v_1.2 in the adult dentate gyrus resulted in an ∼50% reduction in BrdU+ cells, compared with control mice injected with AAV2/2-GFP (Fig. 2*B*; *F*_(1,14)_ = 165.989, *p* < 0.001).

### Cav1.2 channels are necessary for survival of young hippocampal neurons, and not for proliferation of neural precursor cells

**Figure 3.**
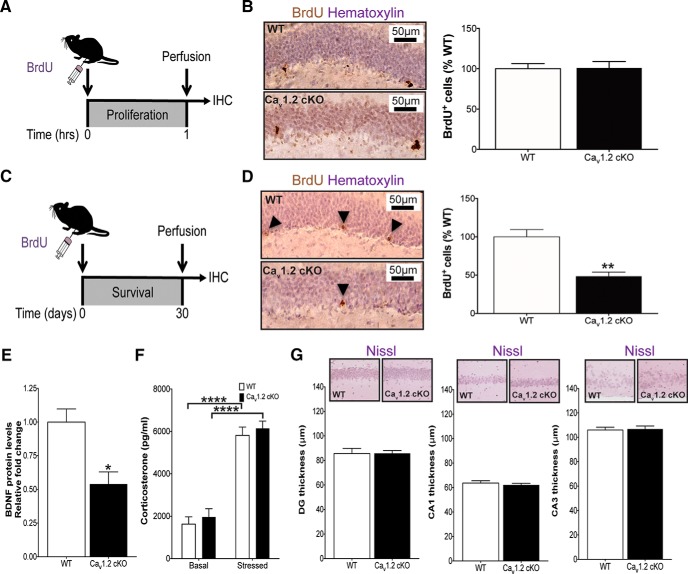
Ca_v_1.2 controls survival of young hippocampal neurons, associated with lower BDNF levels in the absence of differences in corticosterone levels or hippocampus volume. ***A***, ***C***, Graphical representation of BrdU pulse chase experiments to determine proliferation (***A***) versus survival (***C***). IHC, Immunohistochemistry. ***B***, ***D***, Forebrain-Ca_v_1.2 cKO mice display normal proliferation as compared with WT animals, with no difference in BrdU+ cells 1 h after BrdU administration (***B***; WT, *n*=4; KO=3; *p* = 0.935, independent samples *t* test). Forebrain-Ca_v_1.2 cKO mice do, however, show a deficit in survival of young hippocampal neurons, as indicated by significantly lower BrdU+ cells in the dentate gyrus 30 d after BrdU injection (***D***; WT *n*=7; KO, *n*=5; **p = 0.002, independent samples *t* test). Arrows point to BrdU-positive cells. ***E***, BDNF protein levels are significantly lower in forebrain-Ca_v_1.2 cKO mice compared with WT animals (WT, *n*=6; KO, *n*=10; *p = 0.005, independent samples *t* test). ***F***, Corticosterone levels are not different between forebrain-Ca_v_1.2 cKO mice and WT animals (Basal: WT, *n*=14; KO, *n*=15; Stressed: WT, *n*=15; KO, *n*=7; main effect of basal versus stressed ****p < 0.0001; main effect of genotype *p* = 0.4232, two-way ANOVA). ***G***, Nissl staining showed no differences between forebrain-Ca_v_1.2 cKO and WT thickness of the dentate gyrus (DG; *p* = 0.986, independent samples *t* test), CA1 (*p* = 0.518, independent samples *t* test) and CA3 (*p* = 0.898, independent samples Mann–Whitney *U* test) layers of the hippocampus (WT, *n*= 5; KO, *n*= 9). All graphs are represented as mean ± SEM.

The net magnitude of postnatal hippocampal neurogenesis is a balance of proliferation of NPCs and survival of the young hippocampal neurons into which NPCs differentiate, and indeed ∼40% of young hippocampal neurons normally die within the first week of their birth (Pieper et al., 2010). Recently, Ca_v_1.3 has been shown to be essential for both of these processes (Marschallinger et al., 2015). Therefore, we investigated whether Ca_v_1.2 was necessary for proliferation of NPCs, survival of young hippocampal neurons, or both. To address this question, adult forebrain-Ca_v_1.2 cKO mice were injected with a single bolus of BrdU (150 mg/kg, i.p.), followed by transcardial perfusion either 1 h later (to measure proliferation of NPCs; Fig. 3*A*) or 30 d later (to measure survival of young hippocampal neurons; Fig. 3*C*), per established methods (Pieper et al., 2010). We observed no difference in the number of BrdU+ cells at the 1 h time point between forebrain-Ca_v_1.2 cKO mice and wild-type littermates (Fig. 3*B*; *F*_(1,6)_ = 0.039, *p* = 0.935), indicating that in contrast to Ca_v_1.3, Ca_v_1.2 does not affect NPC proliferation. However, forebrain-Ca_v_1.2 cKO mice exhibited an ∼50% lower number of BrdU+ cells relative to wild-type littermates 30 d after BrdU injection (Fig. 3*D*; *F*_(1,11)_ = 18.082, *p* = 0.002), demonstrating that Ca_v_1.2 is necessary for survival of young hippocampal neurons.

### Forebrain-Cav1.2 cKO mice display deficient levels of hippocampal BDNF, with normal glucocorticoid levels and hippocampal size

Because BDNF has been shown to support postnatal hippocampal neurogenesis (Duman and Monteggia, 2006; Chen et al., 2015), and brain levels of BDNF are regulated by L-type calcium channels (Ghosh et al., 1994; Tao et al., 1998), we wondered whether hippocampal levels of BDNF might be altered in forebrain-Ca_v_1.2 cKO mice. Via ELISA, we found that forebrain-Ca_v_1.2 cKO mice have significantly lower hippocampal BDNF protein levels compared with WT littermates (Fig. 3*E*; *F*_(1,15)_ = 11.105, *p* = 0.005).

Next, because glucocorticoid receptors have been shown to modulate connectivity and integration of young hippocampal neurons (Fitzsimons et al., 2013), and forebrain-Ca_v_1.2 cKO mice display markedly high levels of anxiety-like behavior that is often associated with elevated levels of stress hormones in animal models, we wondered whether corticosterone levels might also be altered in forebrain-Ca_v_1.2 cKO mice. Enzyme immunoassay revealed differences in corticosterone levels between basal and stressed groups of each genotype (Fig. 3*F*; *F*_(1,47)_ = 104.1; *p* < 0.001). However, there were no genotype-specific differences in either basal- or stressed-condition corticosterone levels between forebrain-Ca_v_1.2 cKO and WT littermate mice (Fig. 3*F*; *F*_(1,47)_ = 0.6526; *p* = 0.423), demonstrating that lower adult neurogenesis in forebrain Ca_v_1.2 cKO mice is not because of altered corticosterone levels.

Finally, because other mouse models with severe deficits in postnatal hippocampal neurogenesis have been shown to harbor abnormal hippocampal morphology (Pieper et al., 2005), we compared hippocampal morphology in forebrain-Ca_v_1.2 cKO mice with WT littermates. Notably, forebrain-Ca_v_1.2 cKO mice displayed normal overall hippocampal size, as well as normal thickness of the dentate gyrus (*F*_(1,13)_ = 0.022, *p* = 0.986), CA1 (*F*_(1,13)_ = 0.443, *p* = 0.518), and CA3 (*F*_(1,13)_ = 0.056, *p* = 0.898) subregions (Fig. 3*G*).

### P7C3-A20 rescues survival of young hippocampal neurons in forebrain-Cav1.2 cKO mice without affecting BDNF levels

**Figure 4.**
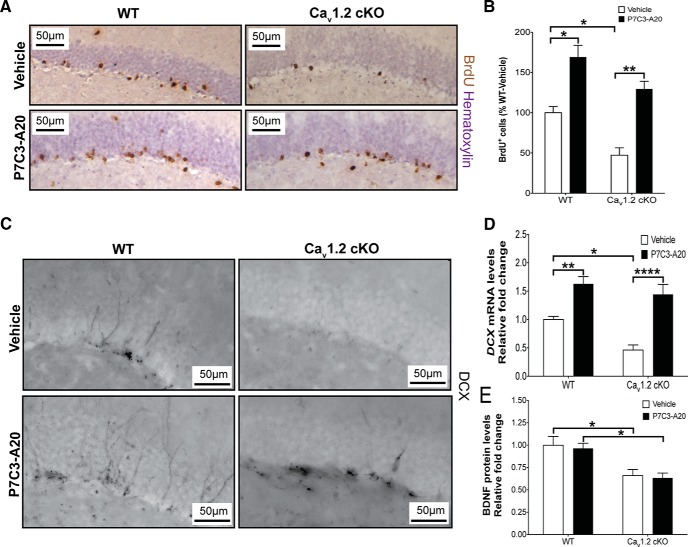
Treatment with P7C3-A20 restores hippocampal neurogenesis in forebrain-Ca_v_1.2 cKO mice without affecting BDNF levels. ***A*–*C***, Treatment with the neuroprotective compound P7C3-A20 significantly increased the levels of BrdU^+^ cells in the dentate gyrus (***A***, ***B***; Veh vs P7C3-A20; ****p < 0.0001; WT-Veh, *n*=3; KO-Veh, *n*=3; WT-A20, *n*=3; KO-A20, *n*=3), DCX protein levels using immunohistochemistry (***C***), and mRNA levels (***D***; Veh: WT vs KO; *p = 0.029; WT: Veh vs P7C3-A20, **p = 0.005; KO: Veh vs P7C3-A20; ****p < 0.0001; WT-Veh, *n*=8; KO-Veh, *n*=7; WT-A20, *n*=9; KO-A20, *n*=8) of both WT and forebrain-Ca_v_1.2 cKO adult animals compared to vehicle-treated groups. ***E***, P7C3-A20 had no effect on BDNF protein levels in either group (WT: Veh vs P7C3-A20, *p* = 0.9996; KO: Veh vs P7C3-A20, *p* > 0.999; WT-VEH, *n*=8; KO-VEH, *n*=5; WT-A20, *n*=8; KO-A20, *n*=6). All graphs are represented as mean ± SEM.

Recently, the novel aminopropyl carbazole P7C3-class of compounds has been discovered and characterized in *in vivo* models of neuron cell death, including protection of young hippocampal neurons that thereby increases the net magnitude of postnatal hippocampal neurogenesis (Pieper et al., 2010, 2014; Macmillan et al., 2011). Active members of this chemical series have been shown to enhance flux of the nicotinamide adenine dinucleotide (NAD) salvage pathway in normal mammalian cells, and facilitate NAD rebound following doxorubicin exposure (Wang et al., 2014). To date, these compounds have shown neuronal protective efficacy in multiple preclinical models of neuropsychiatric disorders, such as Parkinson’s disease (De Jesus-Cortés et al., 2012, 2015; Naidoo et al., 2014), amyotrophic lateral sclerosis (Tesla et al., 2012), stress-associated depressive-like behavior (Walker et al., 2015), aging-associated cognitive decline (Pieper et al., 2010), peripheral nerve crush injury (Kemp et al., 2015), and traumatic brain injury (Blaya et al., 2014; Dutca et al., 2014; Yin et al., 2014). We therefore wondered whether treatment of forebrain-Ca_v_1.2 cKO mice with P7C3-A20, one of the most highly active agents in the P7C3 series, might restore to normal the net magnitude of hippocampal neurogenesis. Indeed, 1 month treatment with P7C3-A20 starting at weaning age fully restored neurogenesis in forebrain-Ca_v_1.2 cKO mice to WT levels, as determined by BrdU-labeling (Fig. 4*A*,*B*; two-way ANOVA, treatment, *F*_(1,8)_ = 18.99, *p* < 0.001; genotype, *F*_(1,8)_ = 50.97, *p* = 0.002) and levels of doublecortin (Fig. 4*C*,*D*; two-way ANOVA; treatment: *F*_(1,28)_ = 41.84, *p* < 0.001; genotype: *F*_(1,28)_ = 8.568; *p* = 0.007). Notably, treatment with P7C3-A20 had no effect on hippocampal BDNF levels (Fig. 4*E*; two-way ANOVA; treatment: *F*_(1,23)_ = 0.1567, *p* = 0.696; genotype: *F*_(1,23)_ = 18.45, *p* < 0.001). Thus, despite the profound deficit in hippocampal BDNF levels in forebrain-Ca_v_1.2 cKO mice, deficient neurogenesis in this model can still be corrected by BDNF-independent mechanisms.

## Discussion

Here, we demonstrate a previously unidentified role of Ca_v_1.2 in regulating survival of young hippocampal neurons in living mice by studying both forebrain-Ca_v_1.2 cKO mice and viral vector-mediated specific hippocampal elimination of Ca_v_1.2 within young hippocampal neurons in adult WT mice. Our *in vivo* data is consistent with a previous *in vitro* study identifying a role of LTCCs in activity-dependent regulation of adult-derived NPCs *in vitro* (Deisseroth et al., 2004), as well as another recent *in vitro* study demonstrating involvement of LTCCs in survival and maturation of newly generated neurons using a clonal line of NPCs established from adult rat hippocampus (Teh et al., 2014). Given the role of hippocampal neurogenesis in multiple forms of neuropsychiatric disease, our findings provide new insight into the potential role of Ca_v_1.2 in the multiple forms of mental illness in which it has been implicated.

We have observed that in the absence of Ca_v_1.2, young hippocampal neurons die at an accelerated rate of ∼50%. Moreover, even though forebrain-Ca_v_1.2 cKO mice display abnormally high anxiety-like behavior (Lee et al., 2012a), and high corticosterone levels associated with stress are known to reduce hippocampal neurogenesis (Cameron and Gould, 1994; Yu et al., 2010), these mice show normal levels of baseline and stressed brain corticosterone, indicating that their deficit in neurogenesis is not due to secondary effects of abnormally high anxiety.

The observed effect of elimination of Ca_v_1.2 on survival of young hippocampal neurons is in contrast to what was recently described for genetic elimination of Ca_v_1.3, which exerts a more profound effect on hippocampal neurogenesis by regulating both proliferation of NPCs and survival of young hippocampal neurons, resulting in reduced hippocampal size (Marschallinger et al., 2015). Presumably, the differential roles of these two major forms of LTCCs in the brain are related to the fact that within the hippocampal neurogenic niche Ca_v_1.2 is expressed exclusively in mature (NeuN-positive cells) young hippocampal neurons, whereas Ca_v_1.3 is expressed in both newly formed immature NPCs (nestin-positive cells) and mature young hippocampal neurons (Marschallinger et al., 2015). An interesting question that will be addressed in future studies is whether this is a cell autonomous or non-autonomous effect. The latter is certainly likely, given that Cav1.2 mediates BDNF production, which can be released from cells to act on both secreting and neighboring neurons. The fact that genetic deletion of Ca_v_1.3 also results in diminished hippocampal size (Marschallinger et al., 2015) suggests that Ca_v_1.3 could play a role in both developmental and postnatal neurogenesis. Here, we show that genetic deletion of Ca_v_1.2, by contrast, has no effect on hippocampal size, suggesting that Ca_v_1.2 plays a specific role in regulating survival of young hippocampal neurons in the mature brain rather than during development. Indeed, we have demonstrated an essential role of Ca_v_1.2 in postnatal hippocampal neurogenesis by viral vector-mediated elimination in adult mice. Apparently, under nonpathologic conditions in the adult animals tested, this decreased survival of young hippocampal neurons is not sufficient to reduce hippocampal size. Future experiments in animals under circumstances of increased cellular stress, such as occurs with injury or aging, will help determine whether decreased survival of young hippocampal neurons in this model compromises overall morphology of the dentate gyrus under stressed conditions. Together, these results suggest that dynamic modulation of Ca_v_1.2-mediated signaling in the adult brain might help ameliorate related disease symptoms.

LTCC signaling has been linked to BDNF production in hippocampal neurons (Ghosh et al., 1994), and we report here for the first time that the brains of forebrain-Ca_v_1.2 cKO mice are deficient in hippocampal levels of BDNF. LTCCs serves as a primary Ca^2+^ source of BDNF synthesis via transcriptional regulation of the promoter for *Bdnf* exon IV, which represents the most highly-expressed *bdnf* splice variant (West et al., 2014). Multiple LTCC-activated transcriptional regulators, including CREB, Ca^2+^ response factor (CaRF), and MeCP2, control *bdnf* expression by binding to the promoter of *bdnf* exon IV (Tao et al 1998, 2002,2009; Chen et al., 2003; Chao and Zoghbi, 2009), and we propose that the lack of activation of these factors in the hippocampus results in lower BDNF in the forebrain of Cav1.2 KO mice. BDNF is known to support neurogenesis, but has not proven to be an effective therapeutic agent to date. We show here that extended treatment of forebrain-Ca_v_1.2 cKO mice with the neuroprotective aminopropyl carbazole P7C3-A20 restored hippocampal neurogenesis to normal levels by ameliorating the aberrantly high rate of death of young hippocampal neurons in these mice. This therapeutic effect was achieved without affecting hippocampal BDNF levels, suggesting that P7C3 compounds offer an alternative therapeutic route to restore neurogenesis in a manner that circumvents deficient BDNF signaling through an independent mechanism.

The net magnitude of postnatal hippocampal neurogenesis is a balance of proliferation of NPCs and survival of the ensuing young hippocampal neurons. Future experiments will address the impact of restoring the net magnitude of hippocampal neurogenesis to normal levels in forebrain-Ca_v_1.2 cKO mice, as hippocampal neurogenesis has been linked to anxiety and depression-like behavior, as well as learning and memory. Such behavioral studies will provide important clarification of the relationship between the observed neural changes and risk for pathology-associated behaviors in this model. Finally, our identification of a new role for Ca_v_1.2 in neuronal cell survival may provide new insight and approaches to treating neuropsychiatric disease. Future experiments will examine whether Ca_v_1.2 also serves a selective role in mediating mature neuronal cell death as well. In conclusion, the results of our work may provide new treatment opportunities for patients suffering from neurodegenerative disease, including forms of mental illness associated with neuronal cell death.

## Synthesis

The decision was a result of the Reviewing Editor Lorna Role and the peer reviewers coming together and discussing their recommendations until a consensus was reached. A fact-based synthesis statement explaining their decision and outlining what is needed to prepare a revision is listed below. The following reviewers agreed to reveal their identity: Kevin Bath, Shaoyu Ge

The authors provide findings supporting the idea that conditional deletion of the Cav1.2 channel in mice decreases the survival of post-natal generated hippocampal dentate gyrus granule neurons and results in diminished BDNF expression. The experiments are well done, and the results shed light on our understanding of how postnatally-born neurons develop and integrate into the pre-existing circuits. The authors go further to demonstrate that levels of survival of young, post-natal neurons can be rescued by the neuroprotective agent P7C3-A20, in a BDNF autonomous fashion. The observation of the effects on neuronal survival are compelling and the manuscript is well written. The authors suggest that such disruptions may contribute to the broad array of pathological conditions associated with mutations in the cacna1c gene.

Major Points:

1. The major reservation of the reviewers was with the interpretation of the data in hand. The manuscript (title through discussion) needs to be carefully rewritten to confine the conclusions to pertaining to the survival of young hippocampal neurons. (i.e. not to neural precursor cells)

Specifically, the reviewers argue that with the current experimental conditions are not appropriate for testing effects of CaV1.2 depletion on proliferation or on neural progenitors. The reason for this more reserved set of conclusions is that the study used the CaV1.2 floxed line crossed with the CaMKII cre. As the authors state -CaMKII starts to be active at specific stages in young neurons. It is generally off in progenitors. As such, the conditional depletion of CaV1.2 in CAMKII neurons is relatively restricted to young neurons in the postnatal brain and as such not apropos for assessment of effects on proliferation

The reviewers agree that the authors can and do answer important questions about the survival of young neurons with the experimental paradigm used.

2. The other major issue raised by the reviewers is that the impact of this paper would be significantly enhanced if the authors had carried out behavioral studies to test if P7C3-A20 were sufficient to also rescue behavioral effects of loss of cacna1c on any pathological outcome (for example anxiety). Such studies would bolster the potential clinical-translational significance of the current findings to show that augmentation of survival of young neurons is, or is not, sufficient to rescue behavioral symptoms of disorders associated with mutations in this gene.

If such behavioral data is available or could be added to the manuscript this would substantially increase the impact of the paper. In lieu of this, it is requested that the authors indicate that such behavioral studies would provide important clarification of the relationship between the observed neural changes and risk for pathology-associated behaviors in the animals.

Additional Issues

1)Re figure 1 and associated methods.

The methods pertinent to Figure 1 need clarification. The authors state the procedure for the Q-PCR used GFP as a marker, but figure 1 presents data from Cav1.2/CamKII-cre mice without GFP. The methods for obtaining the data in Fig 1D should be clarified.

It is surprising that the authors did not observe any changes in the granule cell layer given the strong effects on survival (Fig. 1C&amp;D). It would be helpful for the authors to briefly discuss this apparent discrepancy.

Minor: add scale for figure 1a.

2)Re figure 2.

Please specify which AAV serotype was used to deliver Cre (it appears that most mossy cells and some hilar interneurons got infected?).

The authors unnderscore the cell autonomous effect: to support this conclusion they should confirm that some GFP+ (AAV-Cre-gfp) cells are also DCX positive.

It would be best if the possibility for non-cell autonomous effect is at least presented in the discussion.

Do the authors have data addressing the data in Figure 2 using the AAV CaMKII-Cre - this would better parallel figure 1 studies)

3)Re figure 3.

The presentation of this figure and the text should be revised to reflect the limitations as per major point #1 (above). Since the CaV1.2 gene expression would be expected to be largely intact in the progenitors one would not predict changes in this population (Figure 3a).

The authors should briefly discuss 'why' the BDNF decreases in the CaV1.2-CaMKII off mice.

4)Re Figure 4.

It should be discussed that P7C3-A20 and the CaV1.2 effects could be independent.

** additional note ** One of the authors holds patents on the P7C3 class of neuroprotective chemicals.
